# Intake of different types of red meat, poultry, and fish and incident colorectal cancer in women and men: results from the Malmö Diet and Cancer Study

**DOI:** 10.1080/16546628.2017.1341810

**Published:** 2017-07-18

**Authors:** Alexandra Vulcan, Jonas Manjer, Ulrika Ericson, Bodil Ohlsson

**Affiliations:** ^a^ Division of Internal Medicine, Department of Clinical Science, Lund University, Skåne University Hospital, Malmö, Sweden; ^b^ Division of Surgery, Department of Clinical Science, Lund University, Skåne University Hospital, Malmö, Sweden; ^c^ Diabetes and Cardiovascular Disease, Genetic Epidemiology, Lund University, Skåne University Hospital, Malmö, Sweden

**Keywords:** BMI, colorectal cancer, colon cancer, rectal cancer, sex, red meat

## Abstract

***Background***: Colorectal cancer (CRC) is considered one of the most common forms of cancer in the Western world. High intake of red and processed meat is considered to increase CRC development.

***Objective***: This study examined associations between intake of red meats, poultry, and fish and incident CRC, and if weight status modifies the associations.

***Design***: In the Malmö Diet and Cancer Study, dietary data was collected through a modified diet history method. Via the Swedish Cancer Registry, 728 cases of CRC were identified during 428 924 person-years of follow-up of 16 944 women and 10 987 men.

***Results***: Beef intake was inversely associated with colon cancer. However, in men high intake of beef was associated with increased risk of rectal cancer. High intake of pork was associated with increased incidence of CRC, and colon cancer. Processed meat was associated with increased risk of CRC in men. Fish intake was inversely associated with risk of rectal cancer. No significant interactions were found between different types of meat and weight status.

***Conclusions***: Findings suggest that associations between meat intake and CRC differ depending on meat type, sex, and tumor location in the bowel. Weight status did not modify observed associations.

## Introduction

Colorectal cancer (CRC) is estimated to be one of the most common forms of cancer in the Western world [1,2]. In Sweden, it is the fourth most common form of cancer and around 6500 persons per year develop the disease [3].

Several lifestyle factors are considered to be associated with the development of CRC. Higher body mass index (BMI) and waist circumference are positively associated with risk of CRC, independent of location, sex. or geographic area, as seen in a meta-analysis by Ma et al. [4]. Other lifestyle factors, such as diet, are also considered to be associated with risk of CRC. The World Cancer Research Fund and the American Institute for Cancer Research (WCRF/AICR) have concluded that there is convincing evidence that high intakes of red and processed meat are associated with increased risk of CRC [5]. A meta-analysis indicated that the risk increases by 17% per 100 g daily intake of red meat and by 18% per 50 g daily intake of processed red meat [6]. According to the guidelines from the National Food Agency in Sweden, the intake of red meat should not exceed 500 g per week [7].

The risk associations may differ between gender and tumor location in the bowel [8]. In a meta-analysis by Alexander et al. [9], the association between high intake of processed meat and CRC was mainly seen in men. Although rather few studies have examined specific types of red meat in relation to colorectal cancer, a recent meta-analysis (2015) indicate that the associations differ depending on meat subtypes and sub-sites of CRC. High intakes of beef associated with risk of colon cancer, but not with rectal cancer. Lamb intake was suggested to associate with increased risk of CRC. Intake of poultry was not associated with CRC, but the authors concluded that more studies on pork intake are warranted [10]. Findings regarding high fish intake indicate an inverse association with risk of CRC [11].

The primary objective of the present study was to examine if intake of red meat, considering different subgroups, such as beef and pork, unprocessed and processed red meat, fish, and poultry, is associated with incident CRC, colon cancer, and rectal cancer in women and men from the Malmö Diet and Cancer Study (MDCS). Since obesity may promote development of CRC, our second objective was to investigate whether weight status may modify the association between meat intakes and CRC.

## Subjects and methods

The study was approved by the Regional Ethical Review Board in Lund (50–81, 2013/804).

### Subjects

MDCS is a population-based prospective cohort study in Malmö, Sweden. All men and women living in Malmö between 1991 and 1996, born between 1923 and 1950, were invited to participate. Altogether, 28 098 participants completed all of the baseline examinations after having given their written informed consent. Of those having completed the baseline examination, 167 had been diagnosed with CRC before or at baseline examination, and were therefore excluded from the present study.

### Data collection

At baseline, the participants were asked to fill out questionnaires on socioeconomic, lifestyle, and dietary factors. They also recorded their cooked meals and underwent a diet history interview. Weight, height, and waist circumference were measured by trained nurses. Body composition was estimated with a single-frequency bio-impedance methodology (BIA 103, RJL systems, Detroit, USA). Body fat percentage was calculated using an algorithm provided by the manufacturer.

### Dietary data

Dietary data was gathered through a modified diet history method with a seven-day menu book for registration of meals that varied from day to day, most likely lunch and dinner, cold beverages, and nutrient supplements. In addition, the participants were given a 168-item questionnaire for assessment of consumption frequencies and portion sizes of foods that were not covered in the menu book. Finally, a 45-minute interview completed the dietary assessment. The merged data from the above mentioned methods was then coded using the Swedish Food Data Base [12]. The MDC diet assessment method has been described in detail elsewhere [13,14].

The diet analyses were adjusted for the variables called ‘method version’ and ‘season’. Method version was used because altered coding routines of dietary data were introduced in September 1994 in order to shorten the interview time (from one hour to 45 minutes). This resulted in two slightly different method versions, before and after September 1994, but did not have any major influence on ranking of individuals [13]. The variable season was divided into spring, summer, autumn, and winter depending on when in the year the baseline examination was executed. Dietary change in the past (yes, no) was based on the question ‘Have you substantially changed your eating habits because of illness or some other reasons?’ The relative validity of the MDCS method was evaluated in the Malmö Food study 1984–1985 in a sample of Malmö residents, 105 women and 101 men, 50–69 years old. An 18-day weighted food record was used as the reference method, three days every second month during a year [15,16]. The Pearson correlation coefficients, adjusted for total energy, between the reference method and the MDCS method were 0.70/0.74 (carbohydrates), 0.69/0.64 (fats), 0.53/0.54 (proteins), 0.69/0.74 (fibers), 0.70/0.35 (fish), 0.51/0.43 (low fat meat) and 0.80/0.40 (high fat meat), in women and men, respectively.

The following variables for nutrient intake were used in this study: total energy (MJ), non-alcoholic energy (MJ), carbohydrates (percentage of energy (E%)), fat (E%), protein (E%), saturated fat (g), fiber (g), calcium (mg), folate (mg), iron (mg), and zinc (mg). The following daily intakes of foods were used in this study: red meat (g), unprocessed red meat (g) processed red meat (sausages and cured meat) (g), beef (g), pork (g), poultry (g), fish (g), dairy products (portions of milk, yogurt sour milk, and cheese), fruit and berries (g), and sugar-sweetened beverages (g). Red meat was defined as pork, beef and game. Total red meat included both processed and unprocessed red meat. Intakes of pork and beef were mainly based on non-processed meat, as distinction between pork and beef was not possible for all processed meats based on items included in the food questionnaire. The fish variable consisted of both processed and unprocessed fish. Portions, instead of grams, were used in order to analyse the sum of dairy products, because of different water content and because they usually are consumed in different weights. Standard portion sizes from the national Food Agency in Sweden were used to define portions for dairy products [17].

Energy-adjusted variables were obtained by regressing the food intakes on non-alcohol energy intake [18]. Quintiles of the food residuals were used as exposure categories.

### Cancer cases

In the study, 728 cases of CRC were identified from the Swedish Cancer Registry, of which 463 were colon cancer and 265 rectal cancers, during 428 924 person-years of follow-up. Follow-up time was defined as the time from date of enrolment until date of CRC-diagnosis, death, migration, or end of follow-up (31 December 2010), whichever came first. Information on date of death was collected from The Swedish Cause-of-death registry. Mean follow-up was 15.4 years.

### Other variables

Prevalent diabetes diagnosis was determined from self-reported diagnosis, self-reported medication for diabetes, or information from medical data registries indicating a date of diagnosis before inclusion in the MDCS. Incident diabetes diagnosis was obtained either from the Regional Diabetes 2000 register of Scania, the Malmö HbA1C register or the Swedish National Diabetes Register. In the MDCS, 3245 incident cases of diabetes and 1183 prevalent cases of diabetes were found. In all those with diabetes, 185 cases of incident CRC were found.

Age was obtained from personal identification numbers. Smokers were divided into three categories: current smokers; ex-smokers; and non-smokers. Irregular smoking was counted as current smoking. The subjects estimated their physical activity in minutes and the results were divided into quintiles. The level of education was divided into four different categories: ≤ 8 years; 9–10 years; 11–13 years of education; and university degree. Alcohol intake was divided into four categories: zero; < 15 g/d for women and < 20 g/d for men; 15–30 g/d for women and 20–40 g/d for men; and > 30 g/d for women and > 40 g/d for men. Current use of menopausal hormonal replacement therapy (HRT) and regular use of non-steroid anti-inflammatory drugs (NSAID) were divided into non-users and users. BMI was calculated from measured weight and length. BMI was divided into high (≥ 25 kg/m^2^) and normal and low (< 25 kg/m^2^), after the World Health Organization’s classification of overweight [19].

### Statistical methods

The SPSS statistics package (version 22; IBM Corporation, Armonk, USA) was used for all statistical analyses. The food variables were log-transformed (e-log) to normalize the distribution before analysis. A very small amount (0.0001) was added before transformation, to handle zero intakes [20].

The general linear model was used when examining baseline continuous characteristics in the cases and non-cases, and also adjusting for age and sex, when appropriate, and season and method version for the food variables.

The Cox proportional hazard regression model was used when estimating hazard ratios (HR) of incident CRC, colon cancer, and rectal cancer, depending on quintiles of energy-adjusted food intakes (red meat, beef, pork, unprocessed red meat, processed red meat, poultry, and fish). The basic model included adjustments for age, sex (when appropriate), season, and total energy intake. In addition, the full model was also adjusted for level of education, smoking status, alcohol intake, physical activity, BMI, NSAID use, and when appropriate, for current use of HRT. We also performed the multivariate model excluding BMI, since it might be an intermediate between diet and CRC. Additional models were constructed with further adjustments for diabetes (prevalent and incident) or potential dietary confounders found in previous studies (intake of: fiber; protein; saturated fat; calcium; folate; iron; zinc; fruits and vegetables; milk products; and sugar-sweetened beverages) [5].

Spearman’s correlation matrix was used to examine the correlation between energy-adjusted food intakes (total red meat, beef, pork, unprocessed red meat, processed red meat, poultry, and fish). For intakes where a correlation over 0.40 was found (Supplementary Table 1), additional models were constructed with mutual adjustment in the full models.

A test for interaction between sex or BMI status (< 25 and ≥ 25) and dietary intakes with regard to CRC incidence was performed by adding a multiplicative variable (e.g. sex × diet quintile (treated as continuous variables)) to the full model.

In sensitivity analysis, we excluded individuals with a reported dietary change in the past, all forms of prevalent cancer except cervix cancer in situ or incident and prevalent diabetes. All tests were two-sided and statistical significance was assumed at p < 0.05.

## Results

### Baseline characteristics

Altogether, 16 944 (60.7%) women and 10 987 men completed the baseline examinations, after exclusion of individuals with prevalent CRC. The cases of CRC were, compared with non-cases, older, had a larger waist circumference, and a higher BMI (Table 1). They also had a lower intake of calcium, zinc, beef, and dairy products and a higher intake of pork. Fewer cases had a high level of education, but more of them were highly physically active. Diabetes was more common among the cases, especially in men. In women, the use of HRT was higher among the non-cases.

**Table 1.** Baseline characteristics of cases and non-cases of incident colorectal cancer in the Malmö Diet and Cancer Study cohort.

	Non-cases n = 27 203	Cases n = 728	p-value^a^	Non-cases Women n = 16 572	Cases Women n = 372	p-value^a^	Non-cases Men n = 10 631	Cases men n = 356	p-value^a^
Age (years)	58.0 ± 7.6	61.4 ± 7.0	<0.001	57.3 ± 7.9	61.7 ± 7.1	<0.001	59.1 ± 7.0	61.1 ± 6.8	<0.001*
BMI (kg/m^2^)	25.7 ± 4.0	26.4 ± 4.0	0.014	25.4 ± 4.2	26.0 ± 4.3	0.355	26.2 ± 3.5	26.8 ± 3.6	0.006
Waist (cm)	84.0 ± 12.9	87.5 ± 13.6	0.010	77.8 ± 10.5	79.6 ± 11.2	0.185	93.6 ± 10.1	95.8 ± 10.5	<0.001
Body fat (%)	26.8 ± 7.0	26.5 ± 7.2	0.069	30.7 ± 5.0	31.6 ± 4.9	0.428	20.7 ± 5.0	21.2 ± 5.0	0.100
Energy intake (MJ)	9.5 ± 2.7	9.6 ± 2.7	0.535	8.5 ± 2.1	8.4 ± 2.1	0.996	11.1 ± 2.5	10.8 ± 2.8	0.406
Fat intake (E%)	38.3 ± 6.2	38.5 ± 6.3	0.475	37.7 ± 6.1	37.6 ± 6.0	0.937	39.1 ± 6.3	39.4 ± 6.4	0.275
Protein intake (E%)	15.5 ± 2.5	15.3 ± 2.4	0.275	15.6 ± 2.5	15.5 ± 2.4	0.357	15.3 ± 2.5	15.1 ± 2.4	0.517
Protein (g)	84.0 ± 23.4	83.5 ± 22.8	0.154	76.5 ± 18.8	75.2 ± 19.0	0.822	92.1 ± 25.1	92.1 ± 23.1	0.093
Carbohydrate intake (E%)	46.3 ± 6.2	46.3 ± 6.2	0.777	46.6 ± 6.1	47.0 ± 5.9	0.646	45.7 ± 6.3	45.5 ± 6.5	0.399
Saturated fat (g)	40.9 ± 17.5	41.0 ± 17.4	0.434	36.6 ± 14.6	36.0 ± 13.9	0.824	47.5 ± 19.5	46.2 ± 19.1	0.408
Iron (mg)	18.1 ± 11.9	17.5 ± 4.5	0.177	16.7 ± 12.8	12.3 ± 6.6	0.130	20.1 ± 10.0	19.8 ± 9.6	0.778
Zinc (mg)	13.0 ± 6.0	12.5 ± 5.2	0.033	12.2 ± 6.3	11.5 ± 5.1	0.128	14.2 ± 5.4	13.5 ± 5.2	0.108
Calcium (mg)	1144.8 ± 425.7	1097.5 ± 406.8	0.007	1112.2 ± 393.2	1084.9 ± 398.2	0.615	1195. 8 ± 467.4	1107.6 ± 416.1	0.002
Folate (mg)	290.9 ± 307.1	284.6 ± 388.2	0.445^e^	286.8 ± 310.9	265.8 ± 119.0	0.122^e^	297 ± 301.0	304.3 ± 541.3	0.647^e^
Red meat (g)	97.6 ± 51.2	102.1 ± 53.5	0.147^b,e^	80.7 ± 39.0	80.5 ± 38.0	0.439^b,e^	124.0 ± 57.6	124.6 ± 57.9	0.262^b,e^
Red meat (g/MJ)	10.3 ± 4.5	10.6 ± 4.4	0.058^b,e^	9.6 ± 4.3	9.7 ± 4.2	0.369^b,e^	11.3 ± 4.6	11.6 ± 4.4	0.084^b,e^
Beef (g)	24.6 ± 20.6	23.2 ± 18.8	0.021^b,e^	21.1 ± 17.1	18.8 ± 16.3	0.013^b,e^	29.9 ± 24.1	27.7 ± 20.1	0.443^b,e^
Beef (g/MJ)	2.6 ± 2.1	2.5 ± 1.9	0.024^b,e^	2.5 ± 2.0	2.3 ± 1.9	0.013^b,e^	2.8 ± 2.2	2.7 ± 4.6	0.495^b,e^
Pork (g)	39.6 ± 27.8	42.8 ± 28.7	0.007^b,e^	34.3 ± 22.9	36.7 ± 22.4	0.005^b,e^	48.0 ± 32.2	49.2 ± 32.9	0.381^b,e^
Pork (g/MJ)	4.2 ± 2.8	4.5 ± 2.8	0.006^b,e^	4.1 ± 2.7	4.5 ± 2.7	0.005^b,e^	4.4 ± 2.8	4.6 ± 2.9	0.320^b,e^
Unprocessed red meat (g)	59.0 ± 35.3	60.2 ± 36.7	0.130^b,e^	49.6 ± 27.5	48.7 ± 27.1	0.090^b,e^	73.7 ± 40.8	72.2 ± 41.3	0.770^b,e^
Unprocessed red meat (g/MJ)	6.2 ± 3.4	6.4 ± 3.3	0.098^b,e^	6.0 ± 3.2	6.0 ± 3.2	0.085^b,e^	6.8 ± 3.5	6.7 ± 3.4	0.617^b,e^
Processed red meat (g)	38.6 ± 30.5	41.9 ± 32.4	0.124^b,e^	31.1 ± 23.3	31.8 ± 24.3	0.091^b,e^	50.3 ± 36.2	52.4 ± 36.3	0.691^b,e^
Processed red meat (g/MJ)	4.0 ± 2.9	4.3 ± 2.9	0.101^b,e^	3.7 ± 2.7	3.8 ± 2.7	0.086^b,e^	4.5 ± 3.0	4.8 ± 3.0	0.591^b,e^
Poultry (g)	12.1 ± 16.7	12.8 ± 16.9	0.186^b,e^	11.6 ± 15.2	11.8 ± 15.0	0.096^b,e^	13. 1 ± 18.7	13.8 ± 18.6	0.909^b,e^
Poultry (g/MJ)	1.3 ± 1.8	1.4 ± 1.8	0.173^b,e^	1.4 ± 1.9	1.4 ± 1.9	0.094^b,e^	1.2 ± 1.8	1.3 ± 1.8	0.796^b,e^
Fish (g)	42.2 ± 32.2	43.2 ± 31.2	0.516^b,e^	39.5 ± 28.3	40.2 ± 25.9	0.237^b,e^	46.4 ± 37.1	46.3 ± 35.7	0.586^b,e^
Fish (g/MJ)	4.6 ± 3.5	4.7 ± 3.4	0.461^b,e^	4.8 ± 3.5	4.9 ± 3.2	0.611^b,e^	4.3 ± 3.5	4.5 ± 4.3	0.601^b,e^
Fruits and vegetables (g)	375.8 ± 185.3	363.5 ± 182. 5	0.401^b,e^	395.3 ± 183.8	385.4 ± 174.2	0.299^b,e^	345.5 ± 183.6	340.6 ± 188.3	0.847^b,e^
Sugar sweetened beverages (g)	77.3 ± 149.2	81.3 ± 147.5	0.557^b,e^	66.1 ± 126.8	67.7 ± 124.5	0.213^b,e^	94.7 ± 177.3	95.6 ± 167.3	0.787^b,e^
Dairy products (portions^c^)	6.4 ± 3.9	6.2 ± 3.8	0.027^b,e^	6.0 ± 3.4	5.8 ± 3.1	0.743^b,e^	7.1 ± 4.5	6.6 ± 4.3	0.009^b,e^
Alcohol intake (g)	10.7 ± 12.7	11.2 ± 14.4	0.312^b,e^	7.7 ± 8.7	6.4 ± 8.4	0.382^b,e^	15.5 ± 16.0	16.2 ± 17.4	0.122^b,e^
Smoking, ex/current (%)	33.7/28.4	38.3/26.2	0.172	27.8/28.1	28.0/26.9	0.693	43.0/28.8	38.3/26.2	0.220
Education (> 10 years) (%)	31.9	27.3	0.040	30.3	23.9	0.011	34.3	30.9	0.174
Physical activity high (%)^d^	19.9	23.0	0.043	18.7	20.8	0.314	21.8	25.2	0.126
Incident diabetes (%)	11.6	13.7	0.071	9.4	9.4	0.994	14.9	18.3	0.080
Diabetes (All) (%)	15.8	19.6	0.005	12.7	13.4	0.664	20.5	26.1	0.010
Menopausal hormone therapy, women (%)				19.4	15.3	0.047			

^a^Adjusted for sex and age when appropriate. Calculated with the general linear model for continuous values and Chi^2^-test for categorical values.

^b^Adjusted for age, method, and season, and for sex when appropriate

^c^Standard portion sizes of milk, yoghurt, sour milk and cheese from the National Food Agency in Sweden

^d^Physical activity was defined as high when being in the highest quintile of the whole group

*^e^Calculated with log transformed values*

Values are means ± SD or percentages. P < 0.05 was considered statistically significant

### Dietary intake and colorectal cancer

In the full multivariate model, beef intake was inversely associated with risk for CRC in women (HR: 0.65 for highest compared with lowest quintile; 95% CI: 0.45, 0.95; p for trend = 0.046), but not in men, and a borderline interaction between sex and beef intake was seen (p = 0.068) (Table 2). High pork intake was associated with increased incidence for CRC (HR: 1.39 for highest compared with lowest quintile; 95% CI: 1.09, 1.78; p for trend = 0.023). The association was only significant in women (HR: 1.54 for highest compared with lowest quintile; 95% CI: 1.12, 2.15; p for trend = 0.003), but no significant interaction with sex was seen (p = 0.157). The trend for intake of processed red meat was significantly associated with increased risk for CRC in men (HR: 1.23 for highest compared with lowest quintile; 95% CI: 0.87, 1.73; p for trend = 0.023), but not in women, and a borderline interaction was seen (p = 0.062).

**Table 2.** Hazard ratio (HR) of colorectal cancer associated with intakes of different foods in the Malmö Diet and Cancer study cohort.

			All					Women					Men			
			Basic model^a^		Full model^b^			Basic model^a^		Full model^b^			Basic model^a^		Full model^b^	
Median		Cases/person-years	HR	CI	HR	CI	Cases/person-years	HR	CI	HR	CI	Cases/person-years	HR	CI	HR	CI
**Total Red meat (g/day)**																
**1**	**43.6**	**127/86 428**	1.00		1.00		**94/64 056**	1.00		1.00		**33/22 373**	1.00		1.00	
**2**	**69.3**	**152/85 820**	1.19	0.94, 1.51	1.16	0.91, 1.48	**86/57 761**	1.03	0.77, 1.39	1.02	0.76, 1.37	**66/28 060**	1.59	1.04, 2.41	1.52	1.00, 2.32
**3**	**87.9**	**142/86 102**	1.10	0.86, 1.41	1.07	0.84, 1.37	**75/54 421**	0.97	0.71, 1.32	0.95	0.70, 1.30	**67/31 681**	1.45	0.96, 2.21	1.39	0.91, 2.11
**4**	**107.8**	**146/85 610**	1.14	0.90, 1.46	1.09	0.85, 1.40	**61/49 284**	0.91	0.65, 1.26	0.89	0.64, 1.24	**85/36 325**	1.62	1.08, 2.43	1.51	1.00, 2.28
**5**	**146.2**	**161/84 979**	1.33	1.04, 1.69	1.24	0.97, 1.59	**56/38 777**	1.17	0.83, 1.65	1.14	0.81, 1.62	**105/46 203**	1.70	1.14, 2.53	1.55	1.04, 2.33
**p for trend**			0.058		0.203			0.769		0.902			0.025		0.098	
**Beef (g/day)**																
**1**	**0.3**	**174/86 560**			1.00		**102/56 377**	1.00		1.00		**72/30 184**	1.00		1.00	
**2**	**8.0**	**139/84 121**	0.85	0.67, 1.06	0.84	0.67, 1.05	**78/54 288**	0.84	0.62, 1.13	0.83	0.61, 1.13	**61/29 833**	0.85	0.61, 1.20	0.86	0.61, 1.21
**3**	**15.1**	**157/85 252**	0.99	0.79, 1.23	0.98	0.79, 1.23	**88/54 065**	1.03	0.77, 1.38	1.03	0.76, 1.38	**69/31 186**	0.94	0.68, 1.31	0.95	0.68, 1.32
**4**	**24.0**	**140/86 252**	0.89	0.71, 1.12	0.88	0.70, 1.11	**62/52 425**	0.78	0.56, 1.07	0.77	0.56, 1.07	**78/33 827**	1.02	0.74, 1.41	1.01	0.73, 1.40
**5**	**42.6**	**118/86 739**	0.81	0.63, 1.03	0.79	0.62, 1.01	**42/47 127**	0.66	0.45, 0.96	0.65	0.45, 0.95	**76/39 612**	0.93	0.67, 1.29	0.91	0.65, 1.27
**p for trend**			0.226		0.169			0.055		0.046			0.928		0.962	
**Pork (g/day)**																
**1**	**3.9**	**117/87 256**	1.00		1.00		**68/57 804**	1.00		1.00		**49/29 452**	1.00		1.00	
**2**	**14.2**	**157/84 767**	1.32	1.03, 1.68	1.31	1.03, 1.67	**69/52 604**	1.04	0.74, 1.46	1.04	0.74, 1.46	**88/32 163**	1.63	1.15, 2.32	1.61	1.13, 2.29
**3**	**23.7**	**134/85 538**	1.12	0.87, 1.44	1.10	0.86, 1.41	**67/52 784**	1.03	0.73, 1.44	1.02	0.72, 1.44	**67/32 754**	1.21	0.84, 1.76	1.19	0.82, 1.72
**4**	**34.2**	**153/85 826**	1.28	1.00, 1.63	1.25	0.98, 1.60	**79/52 329**	1.25	0.90, 1.74	1.24	0.89, 1.73	**74/33 507**	1.30	0.90, 1.88	1.26	0.87, 1.82
**5**	**55.2**	**167/85 526**	1.45	1.14, 1.85	1.39	1.09, 1.78	**89/48 760**	1.57	1.13, 2.17	1.54	1.12, 2.15	**78/36 766**	1.34	0.93, 1.93	1.26	0.87, 1.82
**p for trend**			0.007		0.023			0.002		0.003			0.555		0.857	
**Unprocessed red meat (g/day)**																
**1**	**21.3**	**141/85 679**	1.00		1.00		**89/60 792**	1.00		1.00		**52/24 888**	1.00		1.00	
**2**	**39.1**	**147/85 967**	1.02	0.81, 1.28	1.00	0.82, 1.22	**82/56 278**	0.99	0.73, 1.34	0.98	0.72, 1.33	**65/29 689**	1.05	0.73, 1.51	1.02	0.71, 1.47
**3**	**52.1**	**150/85 640**	1.07	0.85, 1.35	0.88	0.68, 1.14	**79/53 682**	1.05	0.77, 1.42	1.03	0.76, 1.40	**71/31 959**	1.10	0.77, 1.58	1.06	0.74, 1.52
**4**	**67.0**	**146/85 624**	1.07	0.85, 1.35	0.98	0.80, 1.20	**67/51 105**	0.98	0.71, 1.35	0.96	0.69, 1.33	**79/34 519**	1.17	0.82, 1.67	1.11	0.78, 1.58
**5**	**96.3**	**144/86 014**	1.07	0.84, 1.37	1.08	1.06, 1.09	**55/42 426**	1.06	0.75, 1.49	1.03	0.73, 1.46	**89/43 588**	1.09	0.77, 1.54	1.00	0.70, 1.43
**p for trend**			0.305		0.589			0.524		0.632			0.494		0.863	
**Processed red meat (g/day)**																
**1**	**6.7**	**120/87 060**	1.00		1.00		**65/58 622**	1.00		1.00		**55/28 439**	1.00		1.00	
**2**	**20.7**	**156/86 124**	1.26	0.99, 1.60	1.25	0.98, 1.59	**100/53 716**	1.64	1.19, 2.26	1.62	1.18, 2.24	**56/32 409**	0.87	0.60, 1.26	0.87	0.60, 1.26
**3**	**31.7**	**142/85 707**	1.15	0.90, 1.47	1.13	0.88, 1.45	**75/52 840**	1.27	0.90, 1.78	1.25	0.89, 1.76	**67/32 867**	1.01	0.70, 1.44	1.00	0.70, 1.43
**4**	**44.0**	**154/85 824**	1.23	0.96, 1.56	1.20	0.94, 1.53	**70/51 459**	1.18	0.83, 1.67	1.18	0.83, 1.67	**84/34 366**	1.23	0.87, 1.73	1.20	0.85, 1.69
**5**	**67.0**	**156/84 209**	1.25	0.98, 1.60	1.20	0.94, 1.53	**62/47 647**	1.15	0.80, 1.65	1.13	0.79, 1.62	**94/36 562**	1.28	0.91, 1.80	1.23	0.87, 1.73
**p for trend**			0.041		0.104			0.824		0.887			0.009		0.023	
**Poultry (g/day)**																
**1**	**0.0**	**141/86 418**	1.00		1.00		**43/35 511**	1.00		1.00		**104/50,907**	1.00		1.00	
**2**	**0.0**	**147/85 274**	0.96	0.74, 1.25	0.94	0.72, 1.22	**86/66 149**	0.97	0.64, 1.47	0.97	0.64, 1.46	**50/19 125**	1.11	0.77, 1.62	1.07	0.73, 1.56
**3**	**3.1**	**150/84 483**	1.04	0.82, 1.32	1.02	0.80, 1.31	**77/55 813**	1.13	0.76, 1.68	1.14	0.76, 1.69	**62/19 125**	1.01	0.73, 1.39	0.99	0.72, 1.36
**4**	**16.5**	**146/86 634**	0.96	0.75, 1.23	0.95	0.74, 1.22	**98/59 689**	1.29	0.88, 1.89	1.28	0.87, 1.88	**35/26 945**	0.62	0.42, 0.91	0.62	0.42, 0.91
**5**	**31.9**	**144/86 115**	1.27	1.01, 1.58	1.24	0.99, 1.55	**68/47 120**	1.22	0.82, 1.81	1.21	0.81, 1.81	**105/38 995**	1.34	1.02, 1.76	1.29	0.98, 1.70
**p for trend**			0.110		0.132			0.124		0.128			0.422		0.536	
**Fish (g/day)**																
**1**	**6.2**	**135/85 629**	1.00		1.00		**57/49 962**	1.00		1.00		**78/35 667**	1.00		1.00	
**2**	**23.2**	**150/86 126**	1.00	0.79, 1.26	1.00	0.79, 1.27	**79/54 549**	1.11	0.78, 1.57	1.12	0.79, 1.59	**71/31 578**	0.93	0.67, 1.28	0.93	0.67, 1.28
**3**	**36.2**	**134/86 220**	0.84	0.66, 1.07	0.85	0.67, 1.08	**81/54 109**	1.08	0.76, 1.53	1.09	0.77, 1.54	**53/32 111**	0.65	0.46, 0.93	0.65	0.46, 0.93
**4**	**51.9**	**151/86 070**	0.91	0.72, 1.15	0.91	0.72, 1.16	**85/55 296**	1.02	0.72, 1.45	1.03	0.73, 1.46	**66/30 774**	0.83	0.59, 1.15	0.82	0.59, 1.14
**5**	**81.8**	**158/84 879**	0.90	0.71, 1.14	0.90	0.71, 1.14	**70/50 366**	0.88	0.61, 1.26	0.88	0.61, 1.27	**88/34 513**	0.95	0.70, 1.30	0.93	0.68, 1.28
**p for trend**			0.308		0.282			0.357		0.393			0.609		0.506	

^a^Adjusted for sex (man, woman), age (continuous), method version (before or after September 1994), season (winter, spring, summer, autumn), and total energy (continuous)

^b^Adjusted for sex (man, woman), age (continuous), method version (before or after September 1994), season (winter, spring, summer, autumn), total energy (continuous), education (<8 years, 9–10 years, 11–13 years or university degree), smoking (current, ex, or never), alcohol intake (zero, <15g/d for women and <20 g/d for men, 15–30 g/d for women and 20–40 g/d for men, >30 g/d for women and >40 g/d for men), use of non-steroid anti-inflammatory drugs (yes, no), and physical activity (quintiles of physical activity)

Values are quintile medians, hazard ratios, and 95% confidence intervals. p < 0.05 was considered statistically significant. HR was calculated by using Cox proportional hazard risk model and was adjusted for energy with the residual method.

### Dietary intake and colon cancer

High intake of beef was inversely associated with risk of colon cancer (HR: 0.60 for highest compared with lowest quintile; 95% CI: 0.44, 0.82; p for trend = 0.009) (Table 3). The inverse association was also significant in women (HR: 0.60 for highest compared with lowest quintile; 95% CI: 0.37, 0.96; p for trend = 0.049) and similar tendencies were seen in men (p for trend = 0.069). High intake of pork was associated with increased risk of colon cancer (HR: 1.41 for highest compared with lowest quintile; 95% CI: 1.04, 1.90; p for trend = 0.021). The association was only significant in women (HR: 1.56 for highest compared with lowest quintile; 95% CI: 1.12, 2.15; p for trend = 0.003), but no significant interaction with sex was seen (p = 0.146). We observed a borderline significant association between high intake of processed meat and increased risk of colon cancer in men (HR: 1.23 for highest compared with lowest quintile; 95% CI: 0.80, 1.90; p for trend = 0.053), but no significant interaction with sex was seen (p = 0.127).

**Table 3.** Hazard ratio (HR) of colon cancer associated with intakes of different foods in the Malmö Diet and Cancer Study cohort.

				All							Women							Men					
Quintiles for all				Basic model^a^			Full model^b^				Basic model^a^			Full model^b^, ^c^				Basic model^a^			Full model^b^		
Total Red meat (g/day)			Cases/ person-years	HR	CI		HR	CI		Cases/person-years	HR	CI		HR	CI		Cases/person-years	HR	CI		HR	CI	
**1**	**43.5**	**87/86 036**		1.00			1.00			**65/63 765**	1.00			1.00			**22/22 272**	1.00			1.00		
**2**	**69.2**	**91/85 236**		1.03	0.77,	1.39	1.00	0.74,	1.35	**52/57 427**	0.88	0.60,	1.27	0.87	0.60,	1.26	**39/27 809**	1.40	0.83,	2.36	1.35	0.80,	2.28
**3**	**87.9**	**91/85 628**		1.02	0.76,	1.38	0.99	0.73,	1.34	**50/54 228**	0.90	0.62,	1.31	0.89	0.61,	1.30	**41/31 400**	1.33	0.79,	2.24	1.28	0.76,	2.16
**4**	**107.7**	**94/85 141**		1.09	0.81,	1.46	1.04	0.77,	1.40	**44/49 143**	0.94	0.64,	1.40	0.93	0.63,	1.38	**50/35 997**	1.42	0.86,	2.36	1.34	0.80,	2.24
**5**	**146.0**	**100/84 499**		1.23	0.91,	1.66	1.16	0.85,	1.57	**36/38 648**	1.07	0.70,	1.64	1.06	0.69,	1.63	**64/45 850**	1.55	0.95,	2.54	1.44	0.87,	2.38
**p for trend**				0.255			0.471				0.884			0.941				0.179			0.336		
**Beef (g/day)**																							
**1**	**0.4**	**124/86 051**		1.00			1.00			**70/56 066**	1.00			1.00			**54/29 985**	1.00			1.00		
**2**	**8.0**	**91/83 696**		0.78	0.59,	1.03	0.77	0.59,	1.02	**54/54 090**	0.86	0.60,	1.24	0.83	0.58,	1.21	**37/29 606**	0.69	0.45,	1.04	0.68	0.45,	1.04
**3**	**15.1**	**94/84 767**		0.78	0.66,	1.14	0.86	0.66,	1.13	**58/53 819**	0.93	0.65,	1.33	1.00	0.70,	1.44	**39/30 948**	0.71	0.47,	1.08	0.71	0.47,	1.08
**4**	**24.0**	**88/85 754**		0.81	0.61,	1.07	0.79	0.60,	1.05	**39/52 220**	0.64	0.43,	0.96	0.73	0.49,	1.09	**49/33 534**	0.85	0.58,	1.26	0.84	0.57,	1.24
**5**	**42.6**	**63/86 256**		0.61	0.45,	0.84	0.60	0.44,	0.82	**26/46 999**	0.43	0.27,	0.70	0.60	0.37,	0.96	**37/39 256**	0.59	0.38,	0.90	0.57	0.37,	0.88
**p for trend**				0.015			0.009				0.0003			0.049				0.090			0.069		
**Pork (g/day)**																							
**1**	**3.7**	**80/86 908**		1.00			1.00			**46/57 590**	1.00			1.00			**34/29 318**	1.00			1.00		
**2**	**14.1**	**90/84 160**		1.12	0.83,	1.52	1.12	0.82,	1.52	**40/52 329**	1.04	0.74,	1.46	1.04	0.74,	1.46	**50/31 831**	1.40	0.90,	2.18	1.38	0.89,	2.15
**3**	**23.7**	**82/85 023**		1.01	0.74,	1.39	1.00	0.73,	1.37	**42/52 552**	1.03	0.73,	1.44	1.02	0.73,	1.44	**40/32 471**	1.07	0.67,	1.71	1.05	0.66,	1.68
**4**	**34.2**	**98/85 355**		1.22	0.90,	1.64	1.19	0.88,	1.62	**58/52 171**	1.25	0.90,	1.74	1.24	0.89,	1.73	**40/33 184**	1.07	0.67,	1.71	1.04	0.65,	1.67
**5**	**55.2**	**113/85 077**		1.45	1.08,	1.95	1.41	1.04,	1.90	**61/48 553**	1.57	1.14,	2.17	1.56	1.12,	2.15	**52/36 524**	1.32	0.84,	2.06	1.26	0.80,	1.97
**p for trend**				0.010			0.021				0.002			0.003				0.687			0.885		
**Unprocessed red meat (g/day)**																							
**1**	**21.3**	**98/85 263**		1.00			1.00			**62/60 513**	1.00			1.00			**36/24 750**	1.00			1.00		
**2**	**39.1**	**91/85 432**		0.83	0.61,	1.14	0.85	0.62,	1.17	**50/55 999**	0.76	0.50,	1.16	0.75	0.49,	1.15	**41/29 433**	0.85	0.48,	1.53	0.89	0.50,	1.60
**3**	**52.1**	**91/85 056**		0.85	0.59,	1.22	0.89	0.61,	1.28	**52/53 401**	0.78	0.45,	1.36	0.76	0.43,	1.34	**39/31 654**	0.81	0.45,	1.46	0.90	0.50,	1.64
**4**	**66.9**	**91/85 163**		0.75	0.48,	1.17	0.80	0.51,	1.27	**44/50 929**	0.58	0.27,	1.22	0.56	0.26,	1.21	**47/34 234**	0.83	0.44,	1.58	0.95	0.49,	1.86
**5**	**95.3**	**92/85 609**		0.76	0.40,	1.44	0.83	0.43,	1.60	**39/42 352**	0.57	0.19,	1.70	0.53	0.17,	1.65	**53/43 257**	0.87	0.37,	2.05	1.05	0.43,	2.59
**p for trend**				0.097			0.201				0.358			0.058				0.453			0.835		
**Processed red meat (g/day)**																							
**1**	**6.7**	**76/86 650**		1.00			1.00			**42/58 392**	1.00			1.00			**34/28 258**	1.00			1.00		
**2**	**20.7**	**105/85 682**		1.32	1.32,	1.20	1.32	0.98,	1.78	**73/53 504**	1.89	1.28,	2.79	1.75	1.19,	2.58	**32/32 178**	0.82	0.50,	1.32	0.82	0.51,	1.33
**3**	**31.7**	**86/85 182**		1.07	1.07,	1.14	1.07	0.78,	1.46	**47/52 575**	1.26	0.82,	1.92	1.15	0.75,	1.77	**39/32 607**	0.96	0.60,	1.52	0.97	0.61,	1.53
**4**	**43.8**	**97/85 229**		1.17	0.42,	1.00	1.17	0.86,	1.56	**45/51 226**	1.20	0.78,	1.84	1.12	0.73,	1.73	**52/34 002**	1.22	0.79,	1.88	1.21	0.78,	1.87
**5**	**66.5**	**99/83 776**		1.16	0.35,	1.22	1.16	0.85,	1.58	**40/47 497**	1.09	0.70,	1.71	1.05	0.67,	1.64	**59/36 283**	1.26	0.82,	1.94	1.23	0.80,	1.90
**p for trend**				0.254			0.385				0.712			0.602				0.036			0.053		
**Poultry (g/day)**																							
**1**	**0.0**	**87/85 887**		1.00			1.00			**24/35 345**	1.00			1.00			**63/50 542**	1.00			1.00		
**2**	**0.0**	**96/84 946**		1.33	0.98,	1.79	0.98	0.71,	1.35	**64/65 950**	1.00	0.65,	1.55	0.97	0.62,	1.51	**32/18 997**	0.82	0.46,	1.49	0.84	0.46,	1.52
**3**	**3.0**	**95/84 132**		1.09	0.79,	1.48	0.93	0.64,	1.36	**55/55 680**	0.89	0.48,	1.64	0.85	0.45,	1.58	**40/28 452**	0.84	0.46,	1.51	0.89	0.49,	1.63
**4**	**16.4**	**85/86 169**		1.19	0.88,	1.61	0.73	0.45,	1.18	**62/59 361**	0.60	0.25,	1.44	0.56	0.23,	1.36	**23/26 807**	0.69	0.36,	1.33	0.76	0.39,	1.48
**5**	**31.9**	**100/85 389**		1.20	0.88,	1.63	0.74	0.37,	1.50	**42/46 860**	0.80	0.22,	2.87	0.69	0.18,	2.59	**28/38 530**	0.63	0.26,	1.50	0.70	0.28,	1.74
**p for trend**				0.176			0.292				0.353			0.265				0.265			0.501		
**Fish (g/day)**																							
**1**	**6.1**	**73/85 032**		1.00			1.00			**35/49 778**	1.00			1.00			**38/35 254**	1.00			1.00		
**2**	**23.4**	**91/85 589**		0.86	0.63,	1.18	0.88	0.64,	1.21	**47/54 293**	0.89	0.57,	1.36	0.88	0.57,	1.36	**44/31 296**	0.75	0.42,	1.35	0.76	0.43,	1.37
**3**	**36.2**	**86/85 795**		0.88	0.61,	1.28	0.92	0.63,	1.33	**55/53 869**	0.92	0.51,	1.66	0.90	0.49,	1.65	**31/31 926**	0.75	0.42,	1.34	0.80	0.45,	1.44
**4**	**51.7**	**103/85 634**		0.67	0.42,	1.06	0.70	0.44,	1.13	**60/55 058**	0.56	0.24,	1.30	0.54	0.23,	1.28	**43/30 576**	0.65	0.34,	1.23	0.71	0.37,	1.37
**5**	**81.4**	**110/84 475**		0.73	0.37,	1.42	0.77	0.39,	1.53	**50/50 198**	0.85	0.25,	2.89	0.77	0.22,	2.75	**60/34 276**	0.62	0.26,	1.45	0.69	0.28,	1.67
**p for trend**				0.098			0.173				0.524			0.241				0.149			0.316		

^a^Adjusted for sex (man, woman), age (continuous), method version (before or after September 1994), season (winter, spring, summer, autumn), and total energy (continuous)

^b^Adjusted for sex (man, woman), age (continuous), method version (before or after September 1994), season (winter, spring, summer, autumn), total energy (continuous), education (<8 years, 9–10 years, 11–13 years or university degree), smoking (current, ex or never), alcohol intake (zero, <15g/d for women and <20 g/d for men, 15–30 g/d for women and 20–40 g/d for men, >30 g/d for women and >40 g/d for men), use of non-steroid anti-inflammatory drugs (yes, no), and physical activity (quintiles of physical activity).

^c^Adjusted for current use of hormonal replacement therapy (yes/no)

Values are quintile medians, hazard ratios, and 95% confidence intervals. p < 0.05 was considered statistically significant. HR was calculated by using Cox proportional hazard risk model and was adjusted for energy with the residual method.

### Dietary intake and rectal cancer

High intake of beef was associated with increased risk of rectal cancer in men (HR: 1.82 for highest compared with lowest quintile; 95% CI: 1.02, 3.25, p for trend = 0.028), but not in women (Table 4), and a significant interaction was seen between beef intake and sex (p = 0.025). High intake of fish was inversely associated with risk of rectal cancer in all (HR: 0.59 for highest compared with lowest quintile; 95% CI: 0.38, 0.92, p for trend = 0.025), and the association did not differ depending on sex (p for interaction = 0.597).

**Table 4.** Hazard ratio (HR) of rectal cancer associated with intakes of different foods in the Malmö Diet and Cancer study cohort.

			All							Women							Men					
Quintiles for all			Basic model^a^			Full model^b^				Basic model^a^			Full model^b^, ^c^				Basic model^a^			Full model^b^		
Total Red meat (g/day)		Cases/person-years	HR	CI		HR	CI		Cases/person-years	HR	CI		HR	CI		Cases/person-years	HR	CI		HR	CI	
**1**	**12.0**	**40/85 673**	1.00			1.00			**29/63 513**	1.00			1.00			**11/22 159**	1.00			1.00		
**2**	**24.9**	**61/84 893**	1.56	1.04,	2.34	1.53	1.02,	2.29	**34/57 244**	1.39	0.84,	2.30	1.38	0.83,	2.29	**27/27 650**	1.99	0.98,	4.01	1.91	0.94,	3.85
**3**	**33.8**	**51/85 110**	1.30	0.85,	1.98	1.25	0.82,	1.91	**25/53 883**	1.11	0.64,	1.91	1.09	0.63,	1.89	**26/31 227**	1.73	0.85,	3.51	1.63	0.80,	3.31
**4**	**45.6**	**52/84 644**	1.26	0.82,	1.93	1.20	0.78,	1.84	**17/48 445**	0.82	0.44,	1.53	0.81	0.43,	1.51	**35/35 799**	1.98	0.99,	3.93	1.82	0.91,	3.64
**5**	**69.2**	**61/84 ,071**	1.57	1.04,	2.39	1.45	0.95,	2.22	**20/38 495**	1.39	0.77,	2.49	1.31	0.72,	2.38	**41/45 576**	2.07	1.05,	4.08	1.85	0.93,	3.68
**p for trend**			0.097			0.236				0.746			0.887				0.053			0.142		
**Beef (g/day)**		**Interaction**				0.025																
**1**	**0.4**	**50/85 393**	1.00			1.00			**32/55 706**	1.00			1.00			**18/29 686**	1.00			1.00		
**2**	**8.0**	**48/83 313**	1.00	0.67,	1.50	1.00	0.67,	1.49	**24/53 850**	0.81	0.47,	1.39	0.81	0.47,	1.39	**24/29 464**	1.37	0.74,	3.53	1.38	0.75,	2.55
**3**	**15.1**	**60/84 279**	1.28	0.88,	1.88	1.28	0.87,	1.87	**30/53 519**	1.08	0.65,	1.79	1.08	0.65,	1.79	**30/30 760**	1.64	0.91,	2.96	1.66	0.92,	3.00
**4**	**24.1**	**52/85 349**	1.20	0.81,	1.76	1.18	0.75,	1.75	**23/52 039**	0.86	0.50,	1.48	0.86	0.50,	1.48	**29/33 311**	1.69	0.94,	3.02	1.69	0.94,	3.03
**5**	**42.7**	**55/86 040**	1.28	0.87,	1.90	1.23	0.83,	1.83	**16/46 850**	0.77	0.42,	1.42	0.76	0.41,	1.40	**39/39 190**	1.82	1.03,	3.24	1.82	1.02,	3.25
**p for trend**			0.242			0.266				0.509			0.487				0.025			0.028		
**Pork (g/day)**																						
**1**	**21.3**	**37/86 474**	1.00			1.00			**22/57 359**	1.00			1.00			**15/29 115**	1.00			1.00		
**2**	**39.2**	**67/84 758**	1.77	1.18,	2.64	1.76	0.92,	2.25	**29/52 233**	1.32	0.75,	2.32	1.33	0.75,	2.34	**38/32 355**	2.28	1.25,	4.15	2.26	1.24,	4.13
**3**	**52.3**	**52/84 758**	1.43	0.94,	2.18	1.39	0.69,	1.91	**25/52 403**	1.24	0.70,	2.20	1.24	0.70,	2.21	**27/32 355**	1.59	0.85,	3.00	1.55	0.82,	2.93
**4**	**67.0**	**55/84 853**	1.49	0.98,	2.27	1.43	0.57,	1.86	**21/51 778**	1.06	0.58,	1.93	1.04	0.57,	1.91	**34/33 075**	1.88	1.01,	3.47	1.79	0.97,	3.33
**5**	**95.3**	**54/84 418**	1.58	1.04,	2.41	1.47	0.47,	2.44	**28/48 190**	1.56	0.88,	2.75	1.50	0.84,	2.67	**24/36 288**	1.45	0.76,	2.75	1.34	0.70,	2.56
**p for trend**			0.265			0.439				0.278			0.356				0.649			0.906		
**Unprocessed red meat (g/day)**																						
**1**	**21.2**	**43/84 844**	1.00			1.00			**27/60 282**	1.00			1.00			**16/24 561**	1.00			1.00		
**2**	**38.8**	**56/85 003**	1.48	0.94,	2.33	1.59	1.00,	2.50	**32/55 763**	1.31	0.72,	2.40	1.42	0.77,	2.61	**24/29 240**	1.26	0.67,	2.38	1.23	0.65,	2.33
**3**	**53.3**	**59/84 716**	1.16	0.70,	1.91	1.28	0.76,	2.13	**27/53 175**	1.20	0.56,	2.56	1.35	0.62,	2.92	**32/31 541**	1.63	0.89,	2.98	1.56	0.85,	2.86
**4**	**70.5**	**55/84 710**	1.04	0.58,	1.84	1.19	0.66,	2.16	**23/50 ,640**	1.14	0.43,	3.01	1.31	0.49,	3.56	**32/34 070**	1.52	0.83,	2.78	1.44	0.78,	2.64
**5**	**103.0**	**52/85 102**	0.89	0.40,	1.97	1.07	0.47,	2.45	**16/42 103**	1.23	0.30,	4.93	1.47	0.35,	6.14	**36/42 998**	1.45	0.80,	2.64	1.32	0.72,	2.44
**p for trend**			0.316			0.700				0.656			0.935				0.080			0.195		
**Processed red meat (g/day)**																						
**1**	**6.7**	**44/86 373**	1.00			1.00			**23/58 278**				1.00			**21/28 095**	1.00			1.00		
**2**	**20.7**	**51/85 055**	1.55	0.99,	2.45	1.67	1.06,	2.64	**27/53 024**	1.36	0.75,	2.48	1.45	0.79,	2.65	**24/32 031**	1.99	0.85,	4.69	2.11	0.89,	4.99
**3**	**31.7**	**56/84 840**	1.20	0.72,	2.01	1.34	0.80,	2.26	**28/52 404**	1.27	0.60,	2.70	1.40	0.66,	2.99	**28/32 435**	1.18	0.48,	2.87	1.28	0.52,	3.14
**4**	**43.9**	**57/84 781**	1.28	0.72,	2.28	1.50	0.83,	2.71	**25/50 943**	1.38	0.54,	3.56	1.56	0.60,	4.07	**32/33 839**	1.22	0.49,	3.04	1.39	0.55,	3.53
**5**	**66.6**	**57/83 324**	1.25	0.57,	2.75	1.54	0.68,	3.50	**22/47 314**	1.96	0.52,	7.41	2.25	0.58,	8.70	**35/36 010**	0.99	0.32,	3.01	1.20	0.38,	3.80
**p for trend**			0.592			0.941				0.742			0.972				0.242			0.490		
**Poultry (g/day)**																						
**1**	**0.0**	**60/85 562**	1.00			1.00			**19/35 299**				1.00			**41/50 263**	1.00			1.00		
**2**	**0.0**	**40/84 333**	1.48	0.93,	2.35	1.59	1.00,	2.53	**22/65 530**	1.64	0.85,	3.20	1.80	0.91,	3.55	**18/18 803**	1.49	0.65,	3.39	1.56	0.68,	3.57
**3**	**3.2**	**44/83 503**	1.31	0.78,	2.19	1.46	0.86,	2.46	**22/55 265**	1.95	0.81,	4.69	2.24	0.91,	5.50	**22/28 239**	0.98	0.42,	2.28	1.04	0.45,	2.44
**4**	**16.5**	**48/85 815**	1.12	0.61,	2.05	1.30	0.69,	2.42	**36/59 108**	1.81	0.55,	5.95	2.16	0.64,	7.35	**12/26 707**	0.84	0.35,	2.02	0.93	0.38,	2.27
**5**	**31.9**	**73/85 161**	1.18	0.51,	2.72	1.43	0.60,	3.42	**26/46 762**	3.92	0.72,	21.23	4.96	0.87,	28.2	**47/38 400**	0.65	0.22,	1.93	0.76	0.25,	2.33
**p for trend**			0.851			0.680				0.734			0.476				0.207			0.407		
**Fish (g/day)**																						
**1**	**6.1**	**62/84 902**	1.00			1.00			**22/49 631**	1.00			1.00			**40/35 271**	1.00			1.00		
**2**	**23.4**	**59/85 245**	0.83	0.55,	1.24	0.84	0.56,	1.26	**32/54 088**	1.14	0.62,	2.08	1.14	0.62,	2.09	**27/31 158**	0.66	0.38,	1.17	0.67	0.38,	1.18
**3**	**36.1**	**48/85 385**	0.66	0.43,	1.01	0.67	0.44,	1.02	**26/53 618**	0.92	0.49,	1.73	0.92	0.49,	1.73	**22/31 767**	0.51	0.28,	0.93	0.52	0.28,	0.95
**4**	**51.6**	**48/85 016**	0.65	0.43,	1.00	0.65	0.43,	1.00	**25/54 719**	0.86	0.42,	1.52	0.78	0.41,	1.50	**23/30 297**	0.60	0.34,	1.06	0.61	0.34,	1.09
**5**	**81.4**	**48/83 827**	0.60	0.39,	0.92	0.59	0.38,	0.92	**20/49 908**	0.71	0.36,	1.39	0.67	0.34,	1.32	**28/33 919**	0.55	0.31,	0.98	0.57	0.32,	1.03
**p for trend**			0.024			0.025				0.224			0.171				0.052			0.075		

^a^Adjusted for sex (man, woman), age (continuous), method version (before or after September 1994), season (winter, spring, summer, autumn), and total energy (continuous)

^b^Adjusted for sex (man, woman), age (continuous), method version (before or after September 1994), season (winter, spring, summer, autumn), total energy (continuous), education (<8 years, 9–10 years, 11–13 years or university degree), smoking (current, ex or never), alcohol intake (zero, <15g/d for women and <20 g/d for men, 15–30 g/d for women and 20–40 g/d for men, >30 g/d for women and >40 g/d for men), use of non-steroid anti-inflammatory drugs (yes, no), and physical activity (quintiles of physical activity)

^c^Adjusted for current use of hormonal replacement therapy (yes/no). Values are quintile medians, hazard ratios, and 95% confidence intervals. p < 0.05 was considered statistically significant. HR was calculated by using Cox propoonal hazard risk model and was adjusted for energy with the residual method.

### Complementary models

In the analysis of CRC, colon and rectal cancer, additional adjustments for potential dietary confounders (intake of: fiber; protein; saturated fat; calcium; folate; iron; zinc; fruits and vegetables; milk products; and sugar-sweetened beverages) did not affect the results and were therefore excluded from further analysis (data not shown).

In the analysis of CRC, colon, and rectal cancer, additional mutual adjustments for the correlated food intakes (total red meat – beef; total red meat – pork; total red meat – unprocessed red meat; total red meat – processed red meat; unprocessed red meat – beef; unprocessed red meat – pork), did not affect the results to any major extent (data not shown).

### Dietary intake, colorectal, colon, and rectal cancer, and body mass index

Excluding BMI from the multivariate model gave virtually similar results (data not shown). Moreover, no significant interactions were found between the different types of meat intakes and BMI status (< 25 or ≥ 25) on CRC (Supplementary Table 2).

### Sensitivity analyses

#### Exclusion of subjects with dietary change

When excluding individuals reporting dietary change in the past, the association between intake of pork and CRC was only borderline significant (HR: 1.15 for highest compared with lowest quintile; 95% CI: 0.86, 1.52; p for trend = 0.074). Apart from that, we did not observe any major changes in any of the results.

#### Exclusion of subjects with prevalent cancer

When excluding individuals with any type of prevalent cancer at baseline, except for cervix cancer in situ, the inverse association between intake of beef and colon cancer became significant also in men (HR: 0.56 for highest compared with lowest quintile; 95% CI: 0.36, 0.88; p for trend = 0.035).

#### Exclusion of subjects with prevalent or incident diabetes

The association between high intake of processed red meat and risk of CRC in men, did not remain significant (HR: 1.05 for highest compared with lowest quintile; 95% CI: 0.71, 1.56; p for trend = 0.155) when excluding individuals with diabetes.

## Discussion

In the present study, high intake of pork was associated with increased risk of CRC, and especially with colon cancer in women. In contrast, high intake of beef was associated with decreased risk of colon cancer, whereas it was associated with increased risk of rectal cancer in men. Furthermore, there was a trend for increased risk of CRC with higher intake of processed meat among the men, mainly driven by colon cancer. Fish intake was inversely associated with rectal cancer.

In line with our findings, Bernstein et al. [8] did not observe high intakes of unprocessed red meat to be associated with a substantially increased risk of CRC when recent results from the Nurses’ Health Study and the Health professionals Follow-up Study were pooled. Similarly to the meta-analysis by Alexander et al. [9], our results indicate a positive association between intake of processed red meat and CRC in men, but not in women, whereas other meta-analyses have not shown differing associations depending on sex [21,22]. Previous meta-analyses have also indicated significant associations between high intake of processed meat and colon cancer, but not with rectal cancer [6,8], although Bernstein et al. [8] concluded that they could not find evidence to show that the associations differed with colon or rectal cancers, but that the intake of processed red meat was especially associated with increased risk of distal colon cancer. Few studies have examined subsites of colon malignancies, but comparable findings were seen in a meta-analysis of three earlier prospective studies [23], whereas positive associations with both proximal and distal colon cancer were seen in a Norwegian study [24]. In a recent meta-analysis (2015) comparing different meat subtypes, high intakes of beef and lamb, but not pork and poultry, were associated with increased risk of CRC [10]. However, when excluding one of the included cohorts due to heterogeneity between the studies, meta-analysis of four prospective studies indicated, similar to our results, an increased risk of CRC at high intakes of pork [10].

The diverse findings depending on type of meat, gender, and tumor site may reflect the complexity of colorectal cancer. Proximal colon and distal colon (including rectum) arise from different embryonic tissues. They also serve different functions, and the mucosal properties and microenvironment differ between segments [25]. As the fecal content is degraded by the microbiota and water and minerals are reabsorbed during the colonic passage, the production of short-chain fatty acids and metabolites varies, and fecal content changes its properties, in distal direction. Traditionally, we consider colon cancers as one disease. This may be misleading, since proximal colon cancer more often have microsatellite instability, CpG island methylator phenotype, and *KRAS* mutations, whereas rectal and distal colon cancers more often have chromosomal instability and *TP53* and *APC* mutations [26]. So far, the incidence of distal cancer has been higher than the incidence of proximal cancer [27]. In future studies, subgroup analysis of proximal or distal CRC ought to be done in addition to analysis of colon and rectal cancer, which is more common in epidemiological studies and therefore more comparable.

Associations between meat intake and CRC may differ depending on the type of meat most frequently consumed in a different population, as well as on intake levels. In the southernmost district in Sweden, where participants of the MDCS cohort were enrolled, pork intake is by tradition high. Although the intake data on meat could be considered to be satisfactory, it is worth noting, that the main part of the meat intake was recorded in the MDCS seven-day menu book, and that fewer days are needed to capture intakes of food consumed more frequently, compared to those consumed more seldom, indicating that intake data on pork may be more valid, compared to that on beef. This may partly explain why beef was not found to associate with higher risk of colon cancer in this study, while such association has been observed in other populations [9,10]. On the other hand, difference in meat production, with fewer antibiotics and growth factors used in Sweden, compared with for example USA, may affect the association between beef intake and risk of CRC differently. It is stated that increased risk for CRC starts at an intake of 500 g/week for red meat and for processed meat only by eating it [5]. In the present study, the estimated median intake in the lowest (44 g/day), and the highest (146 g/day) quintile of red meat, was well below, and respectively above, the stated threshold level.

The gender difference in observed associations may have several explanations. The prolonged gastrointestinal transit time in women compared with men may lead to a prolonged exposure of the mucosa to carcinogens [28]. On the other hand, this may be counteracted by higher meat intake in men compared with that in women in the MDCS. If meat has carcinogen-inducing properties in the bowel, a difference in transit time may decrease the difference in risk between genders caused by difference in meat intake.

Several mechanisms may lie behind observed associations between red meats and CRC. One difference between beef and pork is the amount and composition of fat [29]. The generally higher fat concentration in pork may enhance the excretion of bile acids, which may promote tumorigenesis [30,31]. During the passage down the colon, the bile acids are modified and dehydroxylated [25], leading to different properties along the colon mucosa. Thus, the malignancy risk may vary between different colon segments. The heme iron in red meat is another plausible explanation for the association between red meats and CRC [29], as heme iron damages the colon’s lining [32]. However, the inverse association between beef intake and risk of colon cancer in the present study, and the association between high pork intake and increased risk of CRC, do not support this explanation.

Consumption of red meat may result in exposure to carcinogens through cooking methods, such as cooking meat at a high temperature by barbequing or smoking, or through preservation with nitrite. Heterocyclic amines (HCAs), polycyclic aromatic hydrocarbons (PAHs), and N-nitroso compounds (NOCs) are thought to be factors in development of CRC [33,34]. In addition, processed meat often contains a high amount of salt [35] and it has been discussed if salt may be a risk factor for CRC [36].

Previous indications of protective associations with high fish intake have, consistent with our findings, above all been seen for rectal cancer [11]. Intake of marine omega-3 is thought to be inversely associated with CRC [37]. Mechanisms are thought to be the reducing effect omega-3 has on the omega-6 polyunsaturated fatty acid production of eicosanoids, and inhibition of cyclo-oxygenase-2 [38], but when analyzing associations between omega-3 intake and risk of CRC, no clear association was seen [5]. The high content of vitamin D and selenium has also been suggested as potential mechanisms [39]. This may explain the inverse association between fish and rectal cancer seen in the present study.

Obesity is considered to be an established risk factor for CRC [4]. Low grade inflammation and changes in microbiota are associated with obesity and have been discussed as possible causes [4], along with insulin-like growth factor-1 and leptin [40]. The observed associations in this study did not clearly differ between individuals with BMI above or below 25, and exclusion of BMI in the multivariate model did not substantially change our finding, suggesting that weight status does not have any modifying or mediating effects on the associations between meat intakes and CRC. Yet, we cannot exclude modification if further discriminating between overweight and obese in more well-powered studies.

Diabetes may promote development of CRC, and red and processed meats have in studies shown to be associated with increased risk of diabetes [41,42]. Exclusion of patients with diabetes did not significantly affect the association between intake of pork or beef and risk of CRC, but the association between intake of processed red meat and CRC did not remain significant in men and the risk estimate in the highest quintile changed from 1.23 to 1.05. Loss of power may lie behind this observation; especially since diabetes is more common among men (26% were excluded). Future well-powered studies may reveal if the presence of diabetes has any modifying effect.

The strength of the present study is dietary data of high relative validity [13,15]. As it is a large population-based prospective study with long follow-up, selection bias and reverse causation were minimized. We had also extensive information on potential confounding factors and were able to exclude individuals who reported dietary changes in the past. There are several limitations of this study. Power may be a problem in some of the gender specific analyses. Family history of CRC and inflammatory bowel disease, seen as strong risk factors in development of CRC [22], were not included in the study as information on these risk factors were missing. Furthermore, neither information on the source of some of the processed meat, which may have influenced the findings regarding intakes of beef and pork and their association with risk of CRC, nor information about intake of heme iron was available.

Despite adjustments for possible confounders and known risk factors, occurrence of residual confounding cannot be completely excluded and we have not adjusted for multiple testing, since dietary intakes are highly correlated and the analyses could not be treated as independent.

## Conclusion

In conclusion, our findings suggest that type of meat as well as sex and tumor location in the bowel influence associations between meat intake and risk of CRC. The findings support previous studies indicating that high intake of processed meat above all is associated with increased risk of CRC in men and that high intake of pork may be associated with an increased risk of CRC. Beef intake was in contrast to previous observations inversely associated with colon cancer, but in men associated with increased risk of rectal cancer. Fish intake was inversely associated with rectal cancer. Presence of overweight did not seem to have any major impact on the findings.

## Supplementary Material

Suppl-Meat_processed.docx
