# Supplementary material for: Intake of different types of red meat, poultry, and fish and incident colorectal cancer in women and men: results from the Malmö Diet and Cancer Study
Source: Food Nutr Res. 2017 Jul 18;61(1):1341810. doi: 10.1080/16546628.2017.1341810 (PMC5533139; doi:10.1080/16546628.2017.1341810)
Supplement: Suppl-Meat_processed.docx [file zfnr_a_1341810_sm7508.docx]

| *Supplementary table 1. Correlation coefficients between energy adjusted intakes of total red meat, pork, beef, unprocessed red meat, processed red meat, poultry and fish in individuals in the Malmö Diet and cancer cohort.* | | | | | | | |
| --- | --- | --- | --- | --- | --- | --- | --- |
|  | Total red meat | Red meat –Beef | Red meat – pork | Unprocessed red meat | Processed red meat | Poultry | Fish |
| Total red meat | 1.00 | 0.44 | 0.63 | 0.75 | 0.64 | 0.10 | 0.15 |
| Red meat – Beef | 0.44 | 1.00 | 0.08 | 0.50 | 0.12 | 0.01 | 0.05 |
| Red meat – Pork | 0.63 | 0.08 | 1.00 | 0.59 | 0.30 | 0.05 | 0.01 |
| Unprocessed red meat | 0.75 | 0.50 | 0.59 | 1.00 | 0.04 | 0.08 | 0.11 |
| Processed red meat | 0.64 | 0.12 | 0.30 | 0.04 | 1.00 | 0.07 | 0.10 |
| Poultry | 0.10 | 0.01 | 0.05 | 0.08 | 0.07 | 1.00 | 0.05 |
| Fish | 0.15 | 0.05 | 0.11 | 0.11 | 0.10 | 0.05 | 1.00 |
| Analyses performed with Spearman’s correlation matrix | | | | | | | |

| Supplementary table 2. Hazard ratio (HR) for colorectal, colon, or rectal cancer associated with intakes of different foods in low and normal BMI (<25) or high BMI (≥25) in the Malmö Diet and Cancer Study cohort | | | | | | | | | | | | | | | | | | | | | | | | | |
| --- | --- | --- | --- | --- | --- | --- | --- | --- | --- | --- | --- | --- | --- | --- | --- | --- | --- | --- | --- | --- | --- | --- | --- | --- | --- |
|  |  | **CRC** | | | | | | | | **Colon cancer** | | | | | | | | **Rectal cancer** | | | | | | | |
| Quintiles for all |  |  | **BMI<25^a^** | |  |  | **BMI ≥25^a^** | |  |  | **BMI<25^a^** | |  |  | **BMI ≥25^a^** | |  |  | **BMI <25^a^** | |  |  | **BMI ≥25^a^** | | |
| Total Red meat (g/day) | | **Cases/person-years** | **HR** | **CI** |  | **Cases/person-years** | **HR** | **CI** |  | **Cases/ person-years** | **HR** | **CI** |  | **Cases/person-year** | **HR** | **CI** |  | **Cases/**  **person-years** | **HR** | **CI** |  | **Cases/**  **person-years** | HR | CI |  |
| 1 | **43.6** | **61/48877** | 1.00 |  |  | **65/37468** | 1.00 |  |  | **42/48535** | 1.00 |  |  | **45/37236** | 1.00 |  |  | **19/48392** | 1.00 |  |  | **20/37107** | 1.00 |  |  |
| 2 | **69.3** | **70/41450** | 1.32 | 0.91, | 1.93 | **82/44228** | 1.04 | 0.77, | 1.42 | **39/41290** | 1.21 | 0.76, | 1.92 | **51/43907** | 1.07 | 0.74, | 1.56 | **30/41022** | 1.59 | 0.83, | 3.04 | **31/43689** | 0.98 | 0.57, | 1.68 |
| 3 | **87.9** | **56/42201** | 1.18 | 0.80, | 1.74 | **86/43812** | 1.04 | 0.76, | 1.42 | **34/42002** | 0.94 | 0.58, | 1.53 | **58/43512** | 1.11 | 0.76, | 1.62 | **23/41831** | 1.74 | 0.92, | 3.31 | **28/43175** | 1.00 | 0.58, | 1.72 |
| 4 | **107.8** | **52/38016** | 1.06 | 0.71, | 1.58 | **94/47506** | 1.15 | 0.85, | 1.56 | **29/37769** | 0.80 | 0.48, | 1.35 | **64/47276** | 1.24 | 0.86, | 1.80 | **23/37697** | 1.65 | 0.86, | 3.18 | **29/46879** | 0.98 | 0.57, | 1.69 |
| 5 | **146.2** | **54/33947** | 1.26 | 0.84, | 1.87 | **107/50933** | 1.11 | 0.81, | 1.53 | **29/33815** | 1.01 | 0.61, | 1.68 | **72/50701** | 1.10 | 0.74, | 1.64 | **25/33732** | 1.80 | 0.93, | 3.46 | **36/50363** | 1.12 | 0.65, | 1.93 |
| p for trend |  |  | 0.829 |  |  |  | 0.161 |  |  |  | 0.604 |  |  |  | 0.150 |  |  |  | 0.135 |  |  |  | 0.690 |  |  |
| Beef (g/day) |  |  |  |  |  |  |  |  |  |  |  |  |  |  |  |  |  |  |  |  |  |  |  |  |  |
| 1 | **0.3** | **78/42769** | 1.00 |  |  | **96/43630** | 1.00 |  |  | **51/40995** | 1.00 |  |  | **61/46199** | 1.00 |  |  | **15/37429** | 1.00 |  |  | **18/46014** | 1.00 |  |  |
| 2 | **8.0** | **47/41080** | 0.64 | 0.44, | 0.92 | **92/42942** | 0.99 | 0.74, | 1.31 | **26/39813** | 0.56 | 0.35, | 0.91 | **65/44514** | 0.89 | 0.63, | 1.25 | **22/40349** | 0.78 | 0.42, | 1.42 | **32/44121** | 1.27 | 0.74, | 2.17 |
| 3 | **15.1** | **64/41415** | 0.91 | 0.64, | 1.28 | **93/43779** | 0.98 | 0.74, | 1.31 | **36/40325** | 0.83 | 0.54, | 1.28 | **51/43259** | 0.80 | 0.56, | 1.14 | **26/41983** | 1.06 | 0.60, | 1.87 | **30/42954** | 1.51 | 0.90, | 2.54 |
| 4 | **24.0** | **61/39938** | 0.99 | 0.70, | 1.39 | **78/46171** | 0.79 | 0.58, | 1.07 | **39/40499** | 0.94 | 0.62, | 1.44 | **65/43973** | 0.70 | 0.49, | 1.01 | **27/42099** | 1.09 | 0.62, | 1.92 | **28/43581** | 1.05 | 0.60, | 1.84 |
| 5 | **42.6** | **43/39289** | 0.79 | 0.55, | 1.16 | **75/47408** | 0.84 | 0.61, | 1.14 | **20/40780** | 0.51 | 0.30, | 0.87 | **48/44686** | 0.69 | 0.47, | 1.01 | **30/10815** | 1.32 | 0.76, | 2.30 | **36/44543** | 1.26 | 0.72, | 2.18 |
| p for trend |  |  | 0.989 |  |  |  | 0.088 |  |  |  | 0.299 |  |  |  | 0.016 |  |  |  | 0.210 |  |  |  | 0.662 |  |  |
| Pork (g/day) |  |  |  |  |  |  |  |  |  |  |  |  |  |  |  |  |  |  |  |  |  |  |  |  |  |
| 1 | **3.9** | **55/46028** | 1.00 |  |  | **62/41086** | 1.00 |  |  | **29/41019** | 1.00 |  |  | **51/45126** | 1.00 |  |  | **19/48392** | 1.00 |  |  | **19/46785** | 1.00 |  |  |
| 2 | **14.2** | **62/42975** | 1.24 | 0.84, | 1.83 | **95/41738** | 1.41 | 1.04, | 1.91 | **33/40074** | 1.17 | 0.71, | 1.95 | **62/44053** | 1.20 | 0.83, | 1.75 | **30/41022** | 1.35 | 0.73, | 2.48 | **34/44155** | 2.13 | 1.23, | 3.67 |
| 3 | **23.7** | **54/41389** | 1.15 | 0.77, | 1.70 | **79/44049** | 1.02 | 0.72, | 1.42 | **32/40434** | 1.02 | 0.61, | 1.71 | **47/44354** | 0.90 | 0.60, | 1.34 | **23/41831** | 1.34 | 0.73, | 2.47 | **28/42520** | 1.36 | 0.75, | 2.46 |
| 4 | **34.2** | **67/38282** | 1.34 | 0.91, | 1.97 | **86/47460** | 1.19 | 0.86, | 1.64 | **37/40516** | 1.27 | 0.77, | 2.09 | **59/44557** | 1.18 | 0.81, | 1.73 | **23/37697** | 1.44 | 0.79, | 2.63 | **27/43520** | 1.28 | 0.70, | 2.34 |
| 5 | **55.2** | **55/35818** | 1.41 | 0.96, | 2.08 | **112/49597** | 1.45 | 1.06, | 1.98 | **41/40368** | 1.47 | 0.90, | 2.42 | **71/44524** | 1.43 | 0.98, | 2.07 | **25/33732** | 1.33 | 0.72, | 2.48 | **36/44269** | 1.57 | 0.88, | 2.82 |
| p for trend |  |  | 0.065 |  |  |  | 0.060 |  |  |  | 0.154 |  |  |  | 0.070 |  |  |  | 0.410 |  |  |  | 0.987 |  |  |
| Unprocessed red meat (g/day) | |  |  |  |  |  |  |  |  |  |  |  |  |  |  |  |  |  |  |  |  |  |  |  |  |
| 1 | **21.3** | **55/40734** | 1.00 |  |  | **71/38457** | 1.00 |  |  | **39/36536** | 1.00 |  |  | **60/44284** | 1.00 |  |  | **16/36455** | 1.00 |  |  | **24/44137** | 1.00 |  |  |
| 2 | **39.1** | **64/40644** | 1.18 | 0.82, | 1.70 | **82/43761** | 0.96 | 0.71, | 1.31 | **28/40462** | 1.16 | 0.74, | 1.82 | **52/44564** | 0.88 | 0.61, | 1.28 | **23/40387** | 1.22 | 0.65, | 2.27 | **30/44221** | 1.11 | 0.65, | 1.89 |
| 3 | **52.1** | **62/40712** | 1.15 | 0.79, | 1.66 | **91/45051** | 1.14 | 0.84, | 1.53 | **38/42097** | 0.86 | 0.53, | 1.40 | **62/44420** | 1.09 | 0.76, | 1.55 | **25/41847** | 1.73 | 0.96, | 3.10 | **32/44190** | 1.25 | 0.74, | 2.11 |
| 4 | **67.0** | **57/40738** | 1.09 | 0.75, | 1.60 | **93/46618** | 1.04 | 0.76, | 1.41 | **31/42404** | 0.91 | 0.55, | 1.49 | **58/44546** | 1.02 | 0.70, | 1.47 | **27/42213** | 1.45 | 0.79, | 2.67 | **29/4208** | 1.04 | 0.60, | 1.81 |
| 5 | **96.3** | **53/40658** | 1.04 | 0.70, | 1.54 | **97/50044** | 1.07 | 0.78, | 1.47 | **37/41912** | 0.95 | 0.57, | 1.57 | **58/44800** | 1.06 | 0.72, | 1.54 | **29/41772** | 1.23 | 0.65, | 2.34 | **29/44441** | 1.06 | 0.61, | 1.85 |
| p for trend |  |  | 0.924 |  |  |  | 0.444 |  |  |  | 0.621 |  |  |  | 0.505 |  |  |  | 0.457 |  |  |  | 0.837 |  |  |
| Processed red meat (g/day) | |  |  |  |  |  |  |  |  |  |  |  |  |  |  |  |  |  |  |  |  |  |  |  |  |
| 1 | **6.7** | **50/45045** | 1.00 |  |  | **69/41922** | 1.00 |  |  | **34/36460** | 1.00 |  |  | **48/45217** | 1.00 |  |  | **16/36447** | 1.00 |  |  | **25/44914** | 1.00 |  |  |
| 2 | **20.7** | **72/43432** | 1.37 | 0.93, | 2.02 | **84/42600** | 1.05 | 0.85, | 1.82 | **35/40703** | 1.47 | 0.90, | 2.42 | **61/44447** | 1.24 | 0.85, | 1.82 | **24/40631** | 1.21 | 0.65, | 2.25 | **24/44178** | 0.90 | 0.51, | 1.58 |
| 3 | **31.7** | **64/41405** | 1.30 | 0.88, | 1.92 | **78/44179** | 1.08 | 0.70, | 1.54 | **41/41857** | 1.23 | 0.74, | 2.06 | **50/44549** | 1.04 | 0.70, | 1.54 | **23/41520** | 1.40 | 0.76, | 2.57 | **34/43138** | 1.29 | 0.76, | 2.17 |
| 4 | **44.0** | **49/39232** | 1.12 | 0.75, | 1.68 | **105/46605** | 1.30 | 0.93, | 1.97 | **24/42789** | 1.17 | 0.70, | 1.97 | **69/44544** | 1.35 | 0.93, | 1.97 | **27/42689** | 1.05 | 0.55, | 2.01 | **35/44331** | 1.31 | 0.78, | 2.20 |
| 5 | **67.0** | **58/35478** | 1.38 | 0.93, | 2.04 | **98/48605** | 1.11 | 0.83, | 1.81 | **39/41608** | 1.16 | 0.69, | 1.97 | **62/43858** | 1.23 | 0.83, | 1.81 | **30/41387** | 1.69 | 0.93, | 3.07 | **26/43637** | 1.00 | 0.57, | 1.75 |
| p for trend |  |  | 0.393 |  |  |  | 0.137 |  |  |  | 0.875 |  |  |  | 0.215 |  |  |  | 0.133 |  |  |  | 0.421 |  |  |
| Poultry (g/day) |  |  |  |  |  |  |  |  |  |  |  |  |  |  |  |  |  |  |  |  |  |  |  |  |  |
| 1 | **0.0** | **66/43821** | 1.00 |  |  | **81/42535** | 1.00 |  |  | **36/37689** | 1.00 |  |  | **53/44762** | 1.00 |  |  | **16/37590** | 1.00 |  |  | **17/45935** | 1.00 |  |  |
| 2 | **0.0** | **51/40406** | 0.98 | 0.65, | 1.49 | **84/44649** | 0.92 | 0.66, | 1.30 | **35/40555** | 1.06 | 0.62, | 1.80 | **60/44280** | 1.00 | 0.65, | 1.52 | **20/40498** | 0.84 | 0.43, | 1.66 | **36/43935** | 0.80 | 0.44, | 1.46 |
| 3 | **3.1** | **55/40068** | 1.03 | 0.70, | 1.51 | **84/44282** | 1.00 | 0.735, | 1.37 | **40/42404** | 1.00 | 0.61, | 1.66 | **60/43715** | 1.10 | 0.75, | 1.63 | **28/42217** | 1.07 | 0.59, | 1.93 | **27/42875** | 0.78 | 0.44, | 1.38 |
| 4 | **16.5** | **61/42121** | 1.09 | 0.74, | 1.58 | **72/44483** | 0.94 | 0.68, | 1.29 | **24/41944** | 1.05 | 0.64, | 1.72 | **52/44859** | 0.98 | 0.66, | 1.47 | **26/41718** | 1.12 | 0.63, | 2.01 | **28/43755** | 0.93 | 0.55, | 1.59 |
| 5 | **31.9** | **60/38074** | 1.13 | 0.78, | 1.62 | **113/47983** | 1.24 | 0.92, | 1.66 | **38/40819** | 0.98 | 0.60, | 1.60 | **65/44999** | 1.23 | 0.84, | 1.79 | **30/40652** | 1.33 | 0.78, | 2.28 | **36/44665** | 1.30 | 0.81, | 2.09 |
| p for trend |  |  | 0.473 |  |  |  | 0.185 |  |  |  | 0.740 |  |  |  | 0.387 |  |  |  | 0.146 |  |  |  | 0.204 |  |  |
| Fish (g/day) |  |  |  |  |  |  |  |  |  |  |  |  |  |  |  |  |  |  |  |  |  |  |  |  |  |
| 1 | **6.2** | **55/42021** | 1.00 |  |  | **79/43450** | 1.00 |  |  | **25/40379** | 1.00 |  |  | **66/46123** | 1.00 |  |  | **29/40128** | 1.00 |  |  | **31/44321** | 1.00 |  |  |
| 2 | **23.2** | **57/42021** | 0.92 | 0.63, | 1.35 | **93/44010** | 1.03 | 0.76, | 1.39 | **31/40621** | 1.18 | 0.50 | 2.80 | **59/44520** | 1.04 | 0.71 | 1.52 | **24/40504** | 0.79 | 0.46 | 1.36 | **34/44394** | 1.56 | 0.68 | 3.54 |
| 3 | **36.2** | **53/41580** | 0.83 | 0.56, | 1.23 | **81/44579** | 0.87 | 0.64, | 1.18 | **29/40592** | 1.30 | 0.56 | 3.03 | **60/42932** | 0.89 | 0.33 | 1.31 | **21/40428** | 0.67 | 0.38 | 1.18 | **30/44604** | 1.52 | 0.66 | 3.50 |
| 4 | **51.9** | **66/41120** | 0.96 | 0.66, | 1.40 | **85/44813** | 0.80 | 0.58, | 1.09 | **39/40559** | 1.28 | 0.55 | 3.00 | **55/44331** | 0.89 | 0.55 | 1.31 | **20/40518** | 0.66 | 0.38 | 1.20 | **25/44311** | 0.81 | 0.32 | 2.07 |
| 5 | **81.8** | **62/37750** | 1.15 | 0.80, | 1.66 | **96/47080** | 0.91 | 0.59, | 1.10 | **48/40261** | 1.22 | 1.02 | 4.84 | **50/44726** | 0.96 | 0.70 | 1.41 | **25/40104** | 0.79 | 0.45 | 1.37 | **24/43582** | 0.62 | 0.22 | 1.68 |
| p for trend |  |  | 0.496 |  |  |  | 0.050 |  |  |  | 0.050 |  |  |  | 0.882 |  |  |  | 0.338 |  |  |  | 0.162 |  |  |

| **^a^ Adjusted for sex (man, woman), age (continuous), method version (before or after September 1994), season (winter, spring, summer, autumn), total energy (continuous), education (<8 years, 9-10 years, 11-13 years or university degree), smoking (current, ex or never), alcohol intake (zero, <15g/d for women and <20 g/d for men, 15-30 g/d for women and 20-40 g/d for men, >30 g/d for women and >40 g/d for men), use of non-steroid anti-inflammatory drugs (yes, no), and physical activity (quintiles of physical activity)**  **Values are quintile medians, hazard ratios, and 95% confidence intervals. p < 0.05 was considered statistically significant. HR was calculated by using Cox proportional hazard risk model and was adjusted for energy with the residual method.** |
| --- |
